# Mothers Secretor Status Affects Development of Childrens Microbiota Composition and Function: A Pilot Study

**DOI:** 10.1371/journal.pone.0161211

**Published:** 2016-09-19

**Authors:** Paula Smith-Brown, Mark Morrison, Lutz Krause, Peter S. W. Davies

**Affiliations:** 1 Children’s Nutrition Research Centre, Child Health Research Centre, The University of Queensland, Brisbane, QLD, Australia; 2 The University of Queensland Diamantina Institute, Translational Research Institute, The University of Queensland, Brisbane, QLD, Australia; Colorado State University, UNITED STATES

## Abstract

**Background:**

One mechanism by which early life environment may influence long term health is through modulation of the gut microbiota. It is widely accepted that the optimal source of nutrition in early life is breast milk, with Human Milk Oligosaccharides (HMOs) thought to play an important role in nourishing the developing microbiota. However, mothers with inactive secretor genes have altered HMO composition and quantities in their breast milk. In this pilot study we examine the influence of secretor status and breast-feeding on microbiota composition at 2 to 3 years of age.

**Methods:**

37 children and 17 eligible mothers were recruited. Secretor status was determined from blood and saliva samples using hemagglutination inhibition technique and faecal microbiota composition was examined by 16S rRNA gene sequencing.

**Results:**

Secretor status was determined for 28 eligible children with 20 being secretors (S, 71.4%). Eleven of the 17 mothers were secretors (S, 64.7%). Unweighted UniFrac distances were significantly associated with child secretor status (R^2^ = 0.069, p = 0.030) and with mother secretor status in children exclusively breastfed for at least 4 months (R^2^ = 0.167, p = 0.028), suggesting an influence on the presence/absence of microbes, with *Prevotella* not detected in samples from secretor children and children of secretor mothers. In children who were exclusively breast-fed for at least 4 months of life the abundance of the known HMO consumers *Bifidobacterium* were increased in the children of secretor mothers compared to non-secretor mothers. The relative abundance of an OTU related to *Bacteroides plebeius*, a bacterium noted for its capacity to utilise sulphated polysaccharides for growth, was decreased in these children.

**Conclusions:**

Child and mothers’ secretor status have an impact on childrens’ microbiota composition at 2 to 3 years of age.

## Introduction

The concept that early life environment and nutrition are important in programming lifelong health is now well accepted [1]. One mechanism by which early life environment may have long term impacts on immune and metabolic homeostasis and therefore long term health is through the modulation of the intestinal microbiota composition [2]. In both western and non-western populations the cessation of breast-feeding and the introduction of solid foods leads to a transition in the stool microbiota from a community with a relatively high proportional representation of Actinobacteria, towards one more closely resembling an adult-like configuration by 3 years of age, where the proportional representations of the Bacteroidetes and Firmicutes phyla are predominant [3, 4].

It is widely accepted that the optimal source of nutrition in early life is breast milk, with the World Health Organisation recommending exclusive breast-feeding for the first 6 months, and continued breast-feeding with appropriate complementary foods until at least 2 years of age [5]. The important role that breast-feeding has in nourishing the developing gut microbiota is demonstrated by the fact that human milk oligosaccharides (HMOs) are the third most abundant component in human breast milk [6]. Human milk contains mainly neutral oligosaccharides that consist of core structures and fucosylated carbohydrates, but also a small quantity of acidic oligosaccharides, which are carbohydrates that contain sialic acids or sulphate groups [7].

Four groups of neutral HMOs have been identified that correlate with the subject’s Lewis blood type [7], which is defined by the presence or absence of specific fucose containing glycans on the surface of erythrocytes and glycoproteins in secreted fluids [6]. Lewis blood type is genetically determined by the Lewis gene (*fucosyltransferase 3*), which encodes for α1,3/4 fucosyltransferase, and the Secretor gene (*fucosyltransferase 2*), which encodes for α1,2 fucosyltransferase [8]. Worldwide it is estimated that around 20% of individuals have inactive secretor genes (non-secretor) though geographic and racial differences have been reported [9, 10]. The breast milk of non-secretor mothers lack α1,2 fucosyl-oligosaccharides [7, 11] and contain around half of the amount of neutral oligosaccharides compared to the breast milk of mothers with active Secretor genes [7]. The concentrations of acidic oligosaccharides have been shown to be independent of Lewis blood group [7] and as such the relative abundance is greater in the breast milk of non-secretor mothers [12].

It has been shown that among a limited number of gut microbes tested only *Bifidobacterium* and *Bacteroides* were able to consume HMOs as a sole carbon source and grow, but even this ability was variable between Bifidobacterium spp. [6]. The most common species recovered from infants *(B*. *bifidum*, *B*. *longum*, and *B*. *breve*) show vigorous or moderate growth on HMOs as a sole carbon source, while other strains such as *B*. *adolescentis* and *B*. *animals* show no growth [6]. Consequently, it has been shown that breast-fed infants with a secretor mother possess higher relative counts of *Bifidobacterium* and *Bacteroides* in their first 4 months of life, compared to breast-fed infants whose mother are non-secretors [12]. However, very little is known in terms of whether these differences persist beyond the period of breast-feeding.

An individual’s own secretor status has also been shown to be associated with altered microbiota composition and diversity in both humans [13, 14] and animal models [15] presumably via the variations in glycan composition and structure of mucins. In that context, there are differences in mucin composition between wild-type and “non-secretor” mice and the impact of these differences on the composition and function of the gut microbiota can be affected by the polysaccharide content of the diet [15].

With this background, we hypothesize that variation in HMOs associated with maternal secretor status would provide a selective environmental pressure on infants’ microbiota development leading to long term modification of children’s microbiota composition that persists even after breast-feeding has ceased. More specifically, we expect the higher relative abundance of *Bifidobacteria* and *Bacteroides* seen in breast-fed infants from secretor mothers compared to non-secretor mothers in their first 4 month of life would also be seen at 2 to 3 years of life.

## Method

### Study participants

Thirty seven children and 17 of their mothers were recruited from the Feeding Queensland Babies Study (FQBS) cohort between December 2012 and October 2013. Mothers of 20 of the children were excluded due to pregnancy within the previous 12 months which is known to impact on Lewis blood typing [16]. Other exclusion criteria were: pre-existing gastrointestinal and immunodeficiency disease; antibiotic use in the previous 3 months; medications known to impact microbiota use in the previous 4 weeks; and NSAIDS or antacid use in the previous 2 weeks.

The ongoing FQBS recruited first time mothers whose children were born between June 2010 and April 2011 [17]. The aim of FQBS was to assess infant feeding attitudes and behaviours amongst first time mothers in Queensland, Australia. It involved mothers completing a prenatal questionnaire at 7 to 8 months of pregnancy followed by questionnaires at 2, 4, 6, 9 and 12 months after birth. These questionnaires collected data relating to feeding method which was used to classify children. Children who received only breast milk at the 4 month survey were classified as exclusively breast-fed, while children who received both breast milk and infant formula at the 4 month survey where classified as combined fed. Breast-feeding was initiated in all children and therefore a child was classified as formula-fed if they had discontinued breast-feeding before 2 months of age and as such were reported as receiving only infant formula from the 2 months survey onwards.

### Secretor Status

Secretor status was determined from blood and saliva samples using hemagglutination inhibition technique [18] at the Red Cross Blood Bank, Kelvin Grove, Australia. Blood samples were collected at the Royal Children’s Hospital, Herston, Australia by experienced phlebotomists. Saliva was collected from the young children using Salimetrics Children’s Swabs while mothers were asked to spit into a pot. The saliva samples were immediately frozen and subsequently raised to 100C to inactivate the digestive enzymes.

### Microbiota

Faecal samples were collected from a disposable bed pan (or nappy if not toilet trained) at the participant’s homes within 24 hours of the study visit and frozen immediately at -20C. The frozen samples were transported in insulated bags with frozen ice blocks before being transferred to -80C for storage. Faecal DNA extractions, PCR amplification and library construction for bar-coded 16S rRNA gene amplicon sequencing, using the Illumina Mi-Seq platform, was performed following standard operating protocols used by the Australian Centre for Ecogenomics, University of Queensland, Australia (ecogenomic.org). Detailed methods are provided as supporting information (S1 Text).

### Bioinformatics

QIIME 1.9.0 [19] was used for bioinformatic data analysis. QIIME’s pick_open_reference_otus.py workflow was used to generate OTUs using default parameters (97% sequence similarity; Greengenes reference database–version 13 8 [20]; uclust OTU picking method [21]). The resulting OTU table was filtered to remove any OTU with an abundance of less than 0.05% across all samples.

The OTU table was rarefied to the minimum sample count (42629 reads) for calculation of measures of diversity. Species richness was estimated from the rarefied OTU table using Chao1 [22] while Simpson Index [23] and Faith’s Phylogenetic Diversity [24] were used to estimate diversity. Beta diversity was calculated using weighted and unweighted UniFrac distances [25].

### Statistical Analysis

Adonis [26] was employed using QIIME to associate UniFrac distances with secretor status. DESeq2 on the online Calypso platform (Version 5.2 www.cgenome.net/calypso) was used to explore the impact of secretor status on taxa abundance at the phylum, genus and OTU level. DESeq2 is a method for differential analysis of count data which uses shrinkage estimations for dispersions and fold changes to improve stability and enables a more quantitative analysis of differences [27]. All p values were adjusted for multiple testing using the Benjamini-Hochberg procedure [28].

The impact of secretor status on the predicted metagenome was explored using PICRUSt 1.0.0 (Phylogenetic Investigation of Communities by Reconstruction of Unobserved States) [29] and LEfSe (Linear Discriminant Analysis Effect Size) [30] on the online Galaxy interface (http://huttenhower.sph.harvard.edu/galaxy/). PICRUSt was used to predict KEGG (Kyoto Encyclopedia of Genes and Genomes) functional pathway abundance using a closed reference OTU table created in QIIME using filter_otus_from_otu_table.py script and the Greengenes reference database–version 13 8 [20]. LEfSe was used to identify functional pathways differentially expressed by secretor status. LEfSe is an algorithm for identifying features that characterise the differences between 2 or more groups or conditions [30]. Kruskal-Wallis sum-rank test is first applied to detect features with significant differential abundance and subsequently Wilcoxon rank-sum test is used to investigate biological consistency using a set of pairwise tests among sub-classes. Finally, Linear Discriminant Analysis was employed to estimate the effect size of each differentially abundant feature using the default effect size threshold of 2 (log_10_).

### Ethics

This study was approved by The University of Queensland Medical Research Ethics Committee (Approval Number: 2012001155) and the Metro South Hospital and Health Service Human Research Ethics Committee (HREC Ref: HREC/12/QPAH457) in Brisbane, Australia and conducted in accordance with the principles expressed in the Declaration of Helsinki. All participants were provided with written and verbal information and consent forms were signed by the mothers or a legal guardian.

## Results

### Study cohort

The 37 children (female = 16) recruited were aged 2.2 to 3.1 (mean = 2.67) years and the 17 mothers were aged 20.9 to 43.9 (mean = 35.25) years. Secretor status was determined for 30 out of the 37 child participants. One child was found to have the rare Le(a+b+) blood type classifying them as a partial secretor [31] and was therefore excluded. Another child was excluded due to still being breast-fed, leaving 28 children with 20 being secretors (71.4%). Secretor status was determined for all 17 eligible mothers with 11 being secretors (64.7%). The characteristics of the children by child and mother secretor status are detailed in Table 1. The group of children with a non-secretor mother included 2 formula-fed children while the group of children with a secretor mother included no children classified as formula-fed. To ensure this did not influence the reported results all analysis by maternal secretor status was repeated in an ABF (Any Breast-Feeding) subgroup with the 2 formula-fed children excluded. In addition, all analysis by maternal secretor status was also conducted in an EBF (Exclusively Breast-Fed) sub-group, containing only children who had been exclusively breast-fed for at least 4 months. Child characteristics by mother’s secretor status in the ABF and EBF subgroups are detailed in S1 Table. Birth weight was significantly higher in the children with non-secretor mothers (p = 0.026) and remained significantly higher in the ABF sub-group (p = 0.048) but not the EBF subgroup (p = 0.432), as calculated using independent samples T test. Birth weight was not associated with microbiota composition in this cohort as tested by Adonis analysis of the weighted (r^2^ = 0.04, p = 0.856) and unweighted (r^2^ = 0.07, p = 0.384) UniFrac distances.

**Table 1:** Participant characteristics by child and mother secretor status.

	CHILD SECRETOR STATUS					MOTHER SECRETOR STATUS				
	Secretor		Non-secretor			Secretor		Non-secretor		
Independent sample T test										
	n	Mean(S.D)	n	Mean (S.D)	p	n	Mean (S.D)	n	Mean (S.D)	p
Child Age (years)	20	2.77(0.24)	8	2.57 (0.26)	0.066	11	2.69 (0.28)	6	2.87 (0.17)	0.187
Child BMI WHO Z Score	20	0.4(0.9)	8	0.39 (0.77)	0.983	11	0.12 (0.85)	6	0.25 (0.82)	0.771
Income(AUD)	20	119100(41718)	7	113214 (3880)	0.747	11	131545 (4732)	6	103333 (4578)	0.693
Pre-pregnancy BMI (kg/m2)	18	24.47(3.82)	6	24.63 (5.32)	0.936	11	24.01 (3.81)	6	21.56 (1.7)	0.087
% Gestational Weight Gain	17	21.26(10.63)	7	25.86 (14.88)	0.4	10	22.53 (10.41)	5	33.89 (13.2)	0.09
Gestation length (days)	20	273.95(20.86)	8	277.88 (9.935)	0.618	11	267.73 (26.79)	6	278.83 (6.91)	0.341
Birth Weight WHO Z Score	17	0.4(0.97)	7	0.28 (0.67)	0.766	10	-0.07 (1.06)	5	0.88 (0.37)	0.026
Fisher’s Exact Test										
	n	%	n	%	p	n	%	n	%	p
Gender (Male)	12	60	5	62.5	1	6	54.5	4	66.7	1
Anti-biotics last 12 mths	11	55	5	62.5	1	5	45.5	4	66.7	1
Delivery Mode (C Section)	8	50	3	42.9	1	3	33.3	2	40	1
Exclusively Breast Fed	13	86.4	6	75		8	80	3	50	
Mixed Fed	5	26.3	1	12.5		2	20	1	16.7	
Formula Fed	1	5.3	1	12.5		0		2	33.3	
Secretor Child						10	90.9	3	60	0.305

Children’s characteristics by child and mother secretor status. For continuous variables sample size (n), mean, standard deviation (S.D) and independent sample T test p value are presented. For categorical variables number of samples in category (n), percentage of total sample and Fisher’s Exact test p value are presented.

### Microbiota Composition

The effect of the child’s and mother’s secretor status on the child’s faecal microbiota was explored by applying the multivariate method Adonis on the unweighted and weighted UniFrac distances (Table 2). The child’s secretor status explained 6.9% of the variance in the unweighted UniFrac distances (p = 0.030) while maternal secretor status explained 16.7% of the variance in the unweighted UniFrac distances in children who were classified as exclusively breast-fed at 4 months of age (p = 0.028). Secretor status was not found to be associated with microbial community richness (Chao1) or diversity (Simpson Index and Faith’s Phylogenetic Diversity).

**Table 2:** Effect of secretor status on children’s unweighted and weighted UniFrac distances.

		Unweighted UniFrac		Weighted UniFrac	
	Sample size (secretor)	R^2^	p	R^2^	p
Child Secretor Status	28 (20 S)	0.069	0.030	0.023	0.699
Mother’s Secretor Status	17 (11 S)	0.071	0.256	0.104	0.111
Mother’s Secretor Status—ABF	14(10 S)	0.111	0.102	0.116	0.138
Mother’s Secretor Status–EBF	11 (8 S)	0.167	0.028	0.159	0.081

Results of Adonis analysis (total sample size (number of secretors), R^2^ and p value) of association of unweighted and weighted UniFrac distances with child secretor status, mother secretor status, mother secretor status—ABF (Any Breast-Feeding) subgroup, with the 2 formula-fed children excluded, and mother secretor status—EBF (Exclusively Breast-Fed) sub-group, containing only children who had been exclusively breast-fed for at least 4 months.

DESeq2 was used to explore whether a child’s own or mother’s secretor status was associated with altered taxa abundance at the phylum (Actinobacteria, Bacteroidetes, Firmicutes and Proteobacteria), genus and OTU level (S2 Table). Non-secretor children showed a significantly higher abundance of the genus *Prevotella* (Table 3) compared to secretor children. In all 3 feeding groups children with a non-secretor mother had increased abundance of the genus *Prevotella*, *Phascolarctobacterium* and an unspecified genus of the family *Ruminococcaceae* compared to children with a secretor mother (S2 Table). Notably, children of non-secretor mothers in all 3 feeding groups had increased abundance of OTU 545061 and OTU 349786, both assigned to the species *Prevotella copri* (S2 Table).

**Table 3:** Effect of secretor status on *Prevotella* abundance.

				Median *Prevotella* Abundance by Secretor Status	
	Sample size(secretor)	p	pFDR	S	N-S
Child Secretor Status	28 (20 S)	< 0.001	<0.001	0	3
Mother’s Secretor Status	17 (11 S)	< 0.001	<0.001	0	4
Mother’s Secretor Status–ABF	14(10 S)	< 0.001	<0.001	0	4055.5
Mother’s Secretor Status–EBF	11 (8 S)	< 0.001	<0.001	0	8106

Results of DESeq2 analysis (total sample size (number of secretors), DeSeq2 calculated p value, Benjamini-Hochberg adjusted p values (pFDR) and median absolute count of Prevotella) of association of *Prevotella* abundance by child secretor status, mother secretor status, mother secretor status—ABF (Any Breast-Feeding) subgroup, with the 2 formula-fed children excluded, and mother secretor status—EBF (Exclusively Breast-Fed) sub-group, containing only children who had been exclusively breast-fed for at least 4 months.

### Bifidobacteria and Bacteroides levels

Breast-fed infants with secretor mothers have been shown to harbour increased relative abundance of the HMO consumers *Bifidobacteria* and *Bacteroides* compared to breast-fed infants with non-secretor mothers during their first 4 months of life [12]. To test our hypothesis that these differences in the microbiota would persist at age 2 to 3 years we examined only the children who had been exclusively breast-fed for at least 4 months (n = 11), using DESeq2. The abundance of *Bifidobacterium* was significantly higher in children of secretor mothers (n = 8) than non-secretor mothers (n = 3) (p = 0.036) but the abundance of *Bacteroides* was not different (p = 0.592). In contrast to our hypothesis, the exclusively breast-fed children with a secretor mother had a decreased abundance of OTU 352617 (p < 0.001, pFDR = 0.001) assigned to the species *Bacteroides plebeius* and OTU 344154 (p < 0.001, pFDR < 0.001) and OTU 4376964 (p < 0.001, pFDR 0.033) both assigned to the species *Bacteroides uniformis*.

### PICRUSt Predicted Metagenome

LEfSe was used to explore whether a child’s own or mother’s secretor status was associated with differential abundance of KEGG functional pathways in the children’s PICRUSt predicted metagenomes (S3 Table). A child being a non-secretor was associated with a higher abundance of the Human Disease (LDA Effect Size = 2.345, p = 0.028), Arachidonic Acid Metabolism (LDA Effect Size = 2.133, p = 0.022) and Biosynthesis of Siderophore Group Nonribosomal Peptides (LDA Effect Size = 2.276, p = 0.019) KEGG functional pathways.

**Fig 1:**
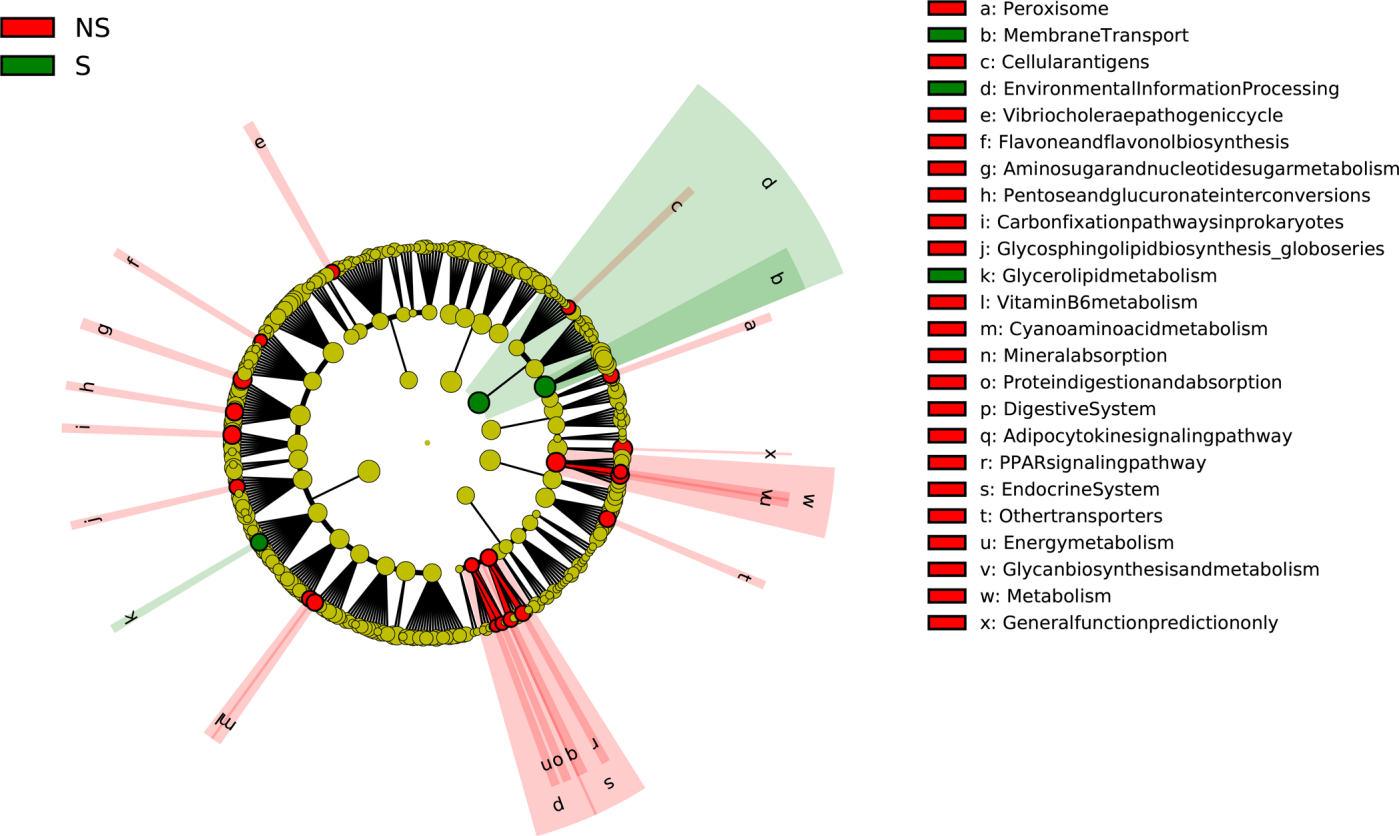
KEGG Functional Pathways differentially abundant by mother’s secretor status. Differentially abundant KEGG Functional Pathways (LDA Effect Size > 2(log10)) in children’s PICRUSt predicted metagenome by mother secretor status (NS = non-secretor mother (n = 6), S = secretor mother (n = 11)), as calculated using LEfSe.

Mothers’ secretor status was associated with altered prevalence of 24 KEGG functional pathways in the children’s PICRUSt predicted metagenome with 21 being increased in the children with non-secretor mothers and 3 being increased in the children with secretor mothers (Fig 1). Performing this analysis in the ABF and EBF sub-groups reduced the number of significantly altered KEGG functional pathways to 19 and 9, respectively. In all 3 feeding groups the predicted metagenome of children with non-secretor mothers were enriched in the abundance of the Peroxisome, Glycosphingolipid Biosynthesis Globo Series, Digestive Systems and Energy Metabolism KEGG functional pathways.

## Discussion

A strength of this study is that data on early life feeding method was collected prospectively therefore avoiding recall bias. A limitation of this study was that secretor status was determined via Lewis blood typing and hemagglutination inhibition technique. This technique does not allow for the differentiation of homozygous and heterozygous Secretor gene expression, which has been shown to further influence microbiota composition and function [32]. In addition, the influence of pregnancy on Lewis blood typing resulted in significant numbers of mothers being excluded in this cohort of 2 to 3 year old children. Despite the small sample size of this pilot study we were able to identify statistically significant variations in the gut microbiota of 2 to 3 year old children that could be linked with the child and mother’s secretor status.

Both child and maternal secretor status were significantly associated with the unweighted UniFrac distances but not with the weighted UniFrac distances, suggesting an influence on presence/absence of microbes. Mothers’ secretor status was significant in explaining a relatively large amount of the variation in the children’s stool microbiota profiles which reached significance only when the analysis was limited to children who had been exclusively breast-fed in their first 4 months of life. This observation suggests that breast-feeding may be an important mechanism by which a mother’s secretor status modulates her child’s gut microbiota.

Lewis et al [12] reported that the abundance of the *Bifidobacterium* and *Bacteroides* genera are greater for infants receiving breast milk from secretor mothers compared to infants receiving non-secretor breast milk during their first 4 months of life. Further analysis revealed a bimodal distribution in *Bifidobacteria* abundance leading the authors to conclude that the reduced *Bifidobacteria* abundance in infants of non-secretor mothers reflected an apparent delay in the transition from low to high *Bifidobacteria* abundance. Here, we show that the microbiota of children who received secretor breast milk exclusively in the first 4 months of life also exhibited higher abundance of *Bifidobacteria* at 2 to 3 years but there were no significant differences observed between groups for *Bacteroides*. According to Freter’s nutrient-niche theory, population sizes of species are determined by the availability of their preferred nutrients [33]. Infant’s gut microbiota are dominated by *B*. *longum*, *B*. *breve* and *B*. *bifidum* which appear specifically adapted to an environment rich in HMOs [6, 33]. In particular, infants with a *Bifidobacteria* dominated microbiota have been shown to have less fucosylated oligosaccharides in their faecal samples compared to infants with *Bacteroides* dominated microbiota, suggesting the former’s preference for fucosylated oligosaccharide consumption [12]. In general terms, bifidobacterial genomes contain fewer genes encoding for the breakdown of complex polysaccharides [34] consistent with their preference for the degradation of HMOs, starch and starch hydrolysates, with the dietary intake of starch at 9 months of age having been shown to be positively correlated with the abundance of *Bifidobacteria* spp. [35]. *Bacteroides* spp. are well recognised for their versatility to utilise a wide variety of complex plant polysaccharides [33] and amino acids [36] and have been identified as being associated with protein intake [36, 37] and the microbiota of populations living Western lifestyles [38–41]. In that context, the altered HMO composition and quantities in non-secretor breastmilk may have negative impacts on *Bifidobacteria* and *Bacteroides* levels during the period of breast-feeding, however the introduction of solid foods and the cessation of breast-feeding would better suit the nutritional versatility and proliferation of *Bacteroides* spp via niche expansion, but not *Bifidobacteria* populations. This is supported by the observed significant increase in *Bacteroides* in the faecal microbiota of exclusively breastfed infants following the introduction of solid foods [42]. Taken together, this could explain why the lack of α1,2 fucosyl-oligosaccharides in non-secretor breast milk appears to result in a sustained reduction in *Bifidobacterium* levels at 2 to 3 years of age, but not *Bacteroides* levels, following the cessation of breast-feeding.

Within the *Bacteroides* genus OTUs related to the species *B*. *plebeius* and *B*. *uniformis* were increased in samples from children who received non-secretor as opposed to secretor breast milk exclusively in their first 4 months of life. The prevalence of *B*. *uniformis* and *B*. *plebeius* within the first 4 months of life has been shown to be associated with both feeding method and genetic risk of Coeliac disease (HLA-DQ genotype), with the high risk genotype being associated with reduced prevalence of both *B*. *uniformis* and *B*. *plebeius* [43]. In addition, non-secretor status is associated with increased risk of some immune-mediated diseases such as Coeliac disease [44] Crohn’s disease [32] and Type 1 Diabetes [45]. Here, we found *Prevotella* and species related to *Prevotella copri* were higher in our non-secretor children and the children of non-secretor mothers, and these bacteria have also been reported to be present at higher levels in untreated Coeliac disease [46] and new onset rhematoid arthritis patients [47] but appear to be reduced in both Crohn’s disease and Type 1 Diabetes [48, 49]. *B*. *plebebius* has been shown to utilise sulphated polysaccharides, and is further distinguished by its acquisition of genes homologous to those in marine algae for this purpose [50] and similarly, some *Prevotella* species have been recognised as being capable of similar de-sulfation of oligosaccharides [51]. These enzymes would plausibly facilitate the utilisation of sulphated gut mucins and acidic sulphated HMOs, whose relative abundance is increased in non-secretor breast milk [12]. Taken together this suggests a mechanism by which non-secretor status would favour the proliferation of these species.

Both the child’s and mother’s secretor status was associated with alterations in the PICRUSt predicted metagenomes of the children. In particular, the non-secretor children were shown to possess relatively greater levels of the Arachidonic Acid Metabolism KEGG functional group, which is consistent with the findings reported by Tong et al [32] for the imputed metagenome of healthy non-secretor adults. Arachidonic acid is known to be involved in inflammatory pathways [52] which is consistent with non-secretor status being associated with inflammatory conditions, such as Crohn’s disease [32].

It has been suggested that lactation has evolved to drive a milk orientated microbiota (MOM) which may play an important role in many of the short and long term benefits of breast-feeding [53]. Here we report that the composition and function of the gut microbiota of 2 to 3 year old children could be linked with the child and mother’s secretor status, presumably mediated by associated alterations in host glycans and breast milk associated HMOs. This highlights the need for mother’s and child’s secretor status to be considered when conducting research into the topic of breast-feeding particularly if the outcome is likely to be related to the microbiota directly or indirectly. In mice, gut microbiota composition can be clustered by secretor status on a standard polysaccharide-rich diet but these specific differences between secretor status were not evident with a polysaccharide deficient diet [15] suggesting complex interactions between the host and dietary sources of polysaccharides on gut microbiota composition. As such, it must be considered that the form and composition of diet in early life would constitute an additional influence guiding the development of the gut microbiota. Understanding these complex interactions is imperative to inform dietary choices in early life to best support long term health and well-being.

## Supporting Information

S1 Table Participant characteristics by child and mother secretor status. Participant characteristics (child’s age, child’s current BMI, family income, mother’s pre-pregnancy BMI, gestational weight gain, gestation length, birth weight, gender, anti-biotic use in previous 12 months, delivery mode, child secretor status and feeding method used in first 4 months of life) presented by child secretor status, mother secretor status, mother secretor status ABF (Any Breast-Feeding in first 4 months of life) subgroup and mother secretor status EBF (Exclusively Breast-Fed in first 4 months of life) subgroup.
(XLSX)

S2 Table Results of DESeq2 analysis. DESeq2 analysis of abundance of taxa at phylum, genus and OTU level in children’s microbiota by child secretor status, mother secretor status, mother secretor status ABF (Any Breast-Feeding in first 4 months of life) subgroup and mother secretor status EBF (Exclusively Breast-Fed in first 4 months of life) subgroup.
(XLSM)

S3 Table Results of LEfSe analysis of PICRUSt predicted metagenome. LEfSe analysis of differential abundance of KEGG Functional Pathways in the childrens’ PICRUSt predicted metagenome by child secretor status, mother secretor status, mother secretor status ABF (Any Breast-Feeding in first 4 months of life) subgroup and mother secretor status EBF (Exclusively Breast-Fed in first 4 months of life) subgroup.
(XLSX)

S1 Text DNA Extraction and Sequencing Methods. (DOCX)
