# Supplementary material for: Mothers Secretor Status Affects Development of Childrens Microbiota Composition and Function: A Pilot Study
Source: PLoS One. 2016 Sep 19;11(9):e0161211. doi: 10.1371/journal.pone.0161211 (PMC5028039; doi:10.1371/journal.pone.0161211)
Supplement: S1 Text — (DOCX) [file pone.0161211.s004.docx]

**S1 Text: DNA Extraction and Sequencing Methods**

**DNA Extraction**

DNA was extracted from 50-100mg of raw sample which was weighed and recorded. An initial step of bead beating using 0.7mm Garnet Bead Tubes (Mo-Bio #13123-50) was performed as per the manufacturer’s instructions. Sample was added into a bead tube filled with 750$\mu l$ of Tissue Lysis Buffer (Promega #A5091). Tubes were vortexed using the Vortex Genie (Mo-Bio #13111, tube adapter #13000-V1-24) for 10 mins at max speed and subsequently centrifuged at 10,000g for 30 seconds (Hereaus Pico). 300μl of the resulting lysate was added into each well of the Promega Maxwell 16 cartridge. DNA extraction was performed using the Maxwell® 16 Research Instrument (Promega) according to the manufacturer’s protocol with the Maxwell 16 Tissue DNA Purification Kit (Promega Cat. # AS1030). DNA was eluted using the SEV setting which is the standard elution volume as outlined in the manufacturer’s instructions. DNA concentration was measured using a Qubit broad range assay (ThermoFisher Scientific; Qubit 3.0 and #Q32853) and was adjusted to a concentration of 5ng/ul.

A quantitative polymerase chain reaction (qPCR) analysis was performed to quantify microbial load and check for inhibitors in the extracted DNA, as follows. The PCR was set up using 5µl of 2X SYBR Green/AmpliTaq Gold DNA Polymerase mix (Life Technologies, Applied Biosystems), 4µl of microbial template DNA and 1µl of primer mix. The 16S 1406F/1525R primer set (0.4µM) was designed to amplify bacterial and archaeal 16S rRNA genes: F - GYACWCACCGCCCGT and R - AAGGAGGTGWTCCARCC. The rpsL F/R primer set (0.2µM), used for inhibition control, amplifies Escherichia coli DH10B only: F - GTAAAGTATGCCGTGTTCGT and R - AGCCTGCTTACGGTCTTTA. Three dilutions 1/100, 1/500 and 1/1000 (microbial template DNA, 16S 1406F/1525R primer set) as well as an inhibition control (E. coli DH10B genomic DNA, rpsL primer set) were run in triplicate for each sample. The PCR was run on the ViiA7 platform (Applied Biosystems) including a cycle of 10 min at 95C (AmpliTaq activation) and 40 cycles of [15s at 95C followed by 20s at 55C and 30s at 72C]. A melt curve was produced by running a cycle of 2 min at 95C and a last cycle of 15s at 60C. The cycle threshold (Ct) values were recorded and analysed using ViiA7 v1.2 software. The extracted DNA was determined to be of sufficient quality to proceed without the need for any further dilution or clean up protocols.

**PCR amplification and Amplicon sequencing**

The 16S rRNA gene encompassing the V6 to V8 regions was targeted using the 926F (5’-AAACTYAAAKGAATTGRCGG-3’) and 1392R (5’-ACGGGCGGTGWGTRC-3’) primers ^1^ modified to contain Illumina specific adapter sequence (926F:5’TCGTCGGCAGCGTCAGATGTGTATAAGAGACAGAAACTYAAAKGAATTGRCGG3’and 1392wR:5’GTCTCGTGGGCTCGGGTCTCGTGGGCTCGGAGATGTGTATAAGAGACAGACGGGCGGTGWGTRC3’). The universal primer pair Univ_SSU_926F-1392wR amplifies the small subunit (SSU) ribosomal RNA of eukaryotes (18S) and prokaryotes (16S) specifically the V6, V7 and V8 regions. In *Escherichia coli*, it amplifies the 926-1392 region of the 16S gene.

Preparation of the 16S library was performed as described, using the workflow outlined by Illumina (#15044223 Rev.B). In the 1st stage, PCR products of ~466bp were amplified according to the specified workflow with an alteration in polymerase used to substitute Q5 Hot Start High-Fidelity 2X Master Mix (New England Biolabs) in standard PCR conditions. Resulting PCR amplicons were purified using Agencourt AMPure XP beads (Beckman Coulter). Purified DNA was indexed with unique 8bp barcodes using the Illumina Nextera XT 384 sample Index Kit A-D (Illumina FC-131-1002) in standard PCR conditions with Q5 Hot Start High-Fidelity 2X Master Mix. Indexed amplicons were pooled together in equimolar concentrations and sequenced on MiSeq Sequencing System (Illumina) using paired end sequencing with V3 300bp chemistry in the Australian Centre for Ecogenomics according to manufacturer’s protocol.

**Quality Control**

After trimming the first 20 bases to remove primer sequences poor quality sequences were removed using a sliding window of 4 bases with an average base quality of 15 using the software Trimmomatic. All reads were then hard trimmed to 250 bases with any less than 250 bases excluded.

**Reference**

1 Engelbrektson, A. *et al.* Experimental factors affecting PCR-based estimates of microbial species richness and evenness. *Isme j* **4**, 642-647, doi:10.1038/ismej.2009.153 (2010).
